# Gene therapy with plasmids encoding IFN-β or IFN-α14 confers long-term resistance to HIV-1 in humanized mice

**DOI:** 10.18632/oncotarget.12512

**Published:** 2016-10-06

**Authors:** Sojan Abraham, Jang-Gi Choi, Nora M. Ortega, Junli Zhang, Premlata Shankar, N. Manjunath Swamy

**Affiliations:** ^1^ Center of Emphasis in Infectious Disease, Department of Biomedical Sciences, Paul L. Foster School of Medicine, Texas Tech University Health Sciences Center, El Paso, TX, USA; ^2^ KM Application Center, Korea Institute of Oriental Medicine, Dong-gu, Daegu, Republic of Korea

**Keywords:** interferon, HIV-1, hydrodynamic injection, Immunology and Microbiology Section, Immune response, Immunity

## Abstract

Because endogenous interferon type I (IFN-I) produced by HIV-1 infection might complicate the analysis of therapeutically administered IFN-I, we tested different humanized mouse models for induction of IFN-I during HIV-1 infection. While HIV-1 induced high levels of IFN-α in BLT mice, IFN-I was undetectable following infection in the Hu-PBL mouse model, in which only T cells expand. We therefore tested the effect of treatment with Pegylated IFN-2 (pegasys), in Hu-PBL mice. Pegasys prevented CD4 T cell depletion and reduced the viral load for 10 days, but the effect waned thereafter. We next expressed IFN-I subsets (IFN-α2, −α6, −α8, −α14, and −β) in Hu-PBL mice by hydrodynamic injection of plasmids encoding them and 2 days later infected the mice with HIV-1. CD4 T cell depletion was prevented in all subtypes of IFN-I-expressing mice by day 10. However, at day 40 post-infection, protection was seen in IFN-β- and IFN-α14-expressing mice, but not the others. The viral load followed an inverse pattern and was highest in control mice and lowest in IFN-β- and IFN-α14-expressing mice until day 40 after infection. These results show that gene therapy with plasmids encoding IFN-β and −α14, but not the commonly used −α2, confers long-term suppression of HIV-1 replication.

## INTRODUCTION

Interferons (IFNs) are a family of cytokines with antiviral, anti-proliferative, and antitumor effects as well as modulatory effects on the innate and adaptive immune responses (reviewed in [1]). IFNs are classified into three types: type I (IFN-α, IFN-β, IFN-ω, IFN-ε, and IFN-κ), type II (IFN-γ), and type III (IFN- λ1, IFN- λ2, and IFN-λ3) [2]. Among the IFN-I, IFN-α and IFN-β have been well studied. IFN-α actually consists of 12 unique subtypes expressed by separate tandemly arranged genes on human chromosome 9, whereas IFN-β exists as a single member [2, 3]. Antibodies specific to different IFN-α subtypes are not available, making studies to test the role of individual subtypes difficult. Because of extensive sequence homology, designing RT-PCR primers is also difficult. For these reasons, the well-studied IFN-α2 subtype is the only one that is currently licensed to treat infections caused by hepatitis B virus (HBV) and HCV [4].

Increased plasma levels of IFN-α and expression of interferon-stimulated genes (ISGs) in peripheral blood mononuclear cells (PBMCs) have been reported in HIV-1-infected patients and SIV-infected monkeys, which suggests that HIV-1 infection induces IFN-I [5–8]. IFN-α has also been tried as a monotherapy or as an adjuvant to antiretroviral therapy during HIV-1 infection but resulted in highly variable results, suggesting that despite its suppressive effect on HIV-1 replication, IFN-α treatment does not significantly reverse CD4 T cell decline or influence clinical outcome [9–14]. Similarly, protective as well as worsening effects of IFN-α were also seen during virulent SIV infection in rhesus macacques [15]. These results, as well as the availability of potent antiretroviral drugs, have reduced enthusiasm for using IFN-α in the clinic. However, interest has re-emerged following the discovery that several HIV-1 restriction factors, such as APOBEC3, MX2, and tetherin, are all ISGs [16]. Moreover, it has also been suggested that achieving a functional HIV-1 cure may be advanced through IFN-α-based therapies [17, 18].

IFN-α2, which was tested in the previously mentioned studies, is the only type 1 IFN approved for therapy. Since antibodies that distinguish different subtypes of IFN-α2 are not available, which subtypes are produced during HIV infection are not known precisely, although it has been reported that IFN-α2 and −α6 subtypes may be more common,[19]. A recent study analyzed the expression of different IFN-α subtypes after exposure of plasmacytoid dendritic cells (pDCs) to HIV-1_BaL_ by a combination of PCR (with primers designed to identify all subtypes) and deep sequencing to identify individual members. The expression of subsets was found to vary, with IFN-α2, −α1/13, and −α10 being the most abundantly expressed subtypes, with −α6 expressed at a 10-fold lower level [20].

All IFN-α subtypes bind to the same type I interferon receptor (IFNAR). However, the binding affinity of different subsets varies [21, 22], resulting in the induction of different signaling pathways and distinct ISG expression patterns [23, 24]. Differences in the efficacy of different subsets have been described in HSV, MCMV, and influenza virus infection in mice, suggesting that these subtypes are not redundant [25, 26]. In HIV-1 infection, an early study suggested that IFN-α2 may be the most potent subtype, but only six IFN-α subtypes were evaluated against an X4-tropic, lab-adapted HIV-1 strain in the MT-2 T cell line [27]. However, Harper et al. recently reported that there was an inverse relationship between IFN-α subtype expression and potency in reducing HIV-1 replication *in vitro*. They found that the most abundantly expressed (upon HIV infection of pDCs) subtypes, IFN-α2 and −α1/13, were much less effective compared with IFN-α6, −α14, and −α8, which were expressed at much lower levels [20].

Testing whether different subsets of IFN-I differentially affect HIV-1 replication *in vivo* is complicated by endogenous IFN-I production elicited by the infection itself. Here we found that endogenous IFN-I is not produced in the Hu-PBL mouse model, allowing assessment of IFN-I subsets for antiviral activity. Moreover, using hydrodynamic injection of plasmids encoding IFN-I subsets, we show that while all subsets suppress HIV-1 replication compared with controls, the effect is longer lasting in IFN-β- and IFN-α14-treated mice, raising the possibility of gene therapy using these plasmids.

## RESULTS AND DISCUSSION

**Figure 1:**
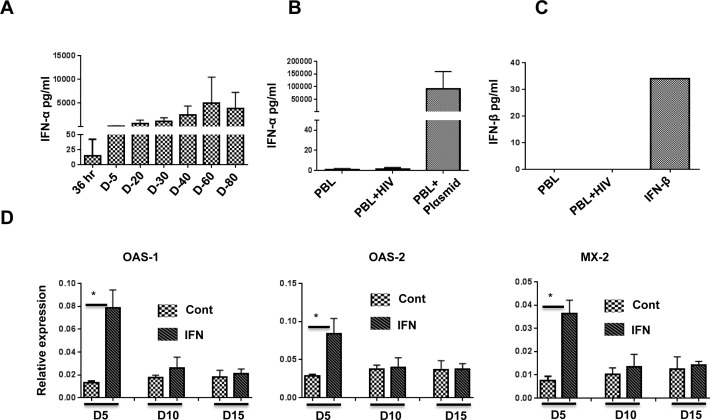
IFN-α is induced after HIV-1 infection in BLT but not Hu-PBL mice, and pegasys treatment in Hu-PBL mice upregulates ISGs **A.**, **B.** Sera of HIV-1-infected BLT mice **A.** and Hu-PBL mice **B.** at the indicated time points were tested for expression of IFN-α by ELISA. In **B.**, PBL refers to uninfected Hu-PBL mice (negative control), PBL + HIV indicates infected mice (test group), and PBL + Plasmid indicates sera from Hu-PBL mice injected with IFN-α-encoding plasmid (positive control). For **A.** and **B.**, *n* = 6 mice/group. **C.** HIV-infected Hu-PBL mice were tested for expression of IFN-β by ELISA. The positive control was soluble IFN-β; *n* = 6 mice. **D.** ISG expression in PBMCs isolated from control and pegasys (200 ng)-injected mice at the indicated time points was tested by qRT-PCR; *n* = 4 mice. Cont, untreated control mice. IFN, pegasys-treated mice. Error bars = 1 SD. Statistical analysis was carried out using non-parametric Mann-Whitney test.

Our aim was to test the efficacy of exogenously administered IFN-I for anti-HIV-1 activity *in vivo*. Since HIV-1 infection itself is expected to induce IFN-I, we sought an animal model in which endogenous IFN-I would not complicate the analysis of results. Therefore, we tested BLT and Hu-PBL mice for production of IFN-I following HIV-1 infection. BLT mice were produced by implanting human fetal thymus and liver tissue under the renal capsule in NOD-scid IL2rγc^null^ (NSG) mice, followed by i.v. injection of autologous HSCs isolated from fetal liver. After confirming reconstitution of human immune cells at 12-15 weeks, the mice were infected with the HIV-1_BaL_ strain (20 ng p24/animal). For the Hu-PBL model, NSG mice were implanted with human PBMCs, and after verifying human T cell reconstitution on day 10, the mice were infected with HIV-1_BaL_. On different days following infection, the sera were tested for IFN-I using the human IFN-α Multi-Subtype and IFN-β ELISA Kit. While a high level of IFN-α was detectable in BLT mice after HIV-1 infection, no IFN-α or −β could be detected in Hu-PBL mice (Figure 1A-1C). This result is probably due to the fact that plasmacytoid dendritic cells (pDCs) are critical for IFN-I production. While pDCs are present in BLT mice, they are not present in Hu-PBL mice, in which only T cells expand due to xenogenic activation. This result is also consistent with the results of Li et al. showing that depletion of pDCs using monoclonal antibodies in BLT mice prior to HIV-1 infection abolished IFN-I production [28]. Since IFN-I was not produced in Hu-PBL mice, this would serve as a good animal model to study the role of IFN-I subsets without complications from HIV-induced IFN-I. We therefore used this model for further studies.

### Pegasys treatment transiently protects against HIV replication in Hu-PBL mice

To determine whether pegylated IFN-α2 (pegasys) can be used in Hu-PBL mice, we tested for upregulation of interferon-stimulated genes (ISGs) following pegasys treatment. To do this, we transplanted human PBMCs into NSG mice, and after verifying human T cell expansion on day 10, treated these mice once with 200 ng of pegasys. Upregulation of select ISGs was tested by qRT-PCR. Compared with control mice, maximal upregulation of ISGs was seen on day 5 and declined to pre-treatment levels by day 15 (Figure 1D). Therefore, we chose 8-10-day intervals for pegasys administration after HIV-1 infection.

**Figure 2:**
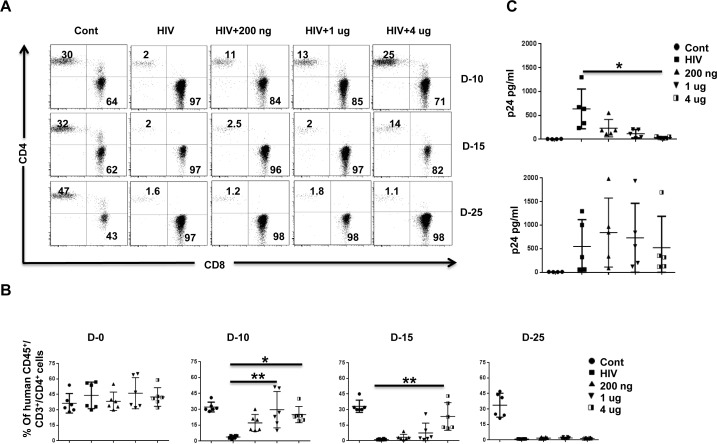
IFN-α2 (pegasys) treatment delays HIV disease progression in Hu-PBL mice **A.**, **B.** Hu-PBL mice were treated with the indicated doses of pegasys and infected 2 days later with HIV-1_BaL_. Pegasys treatment was repeated on days 8 and 18 after infection. Blood collected at the indicated number of days after infection was tested for CD4 T cell depletion by flow cytometry. A representative flow cytometric profile of CD4 and CD8 T cells within the human CD45-gated cell population **A.**, and cumulative data from 6 mice **B.** is shown. In B, each symbol represents an individual mouse. Cont, uninfected Hu-PBL mice; HIV, untreated HIV-1-infected Hu-PBL mice; 200ng, 1ug, 4ug, infected mice treated with the indicated doses of pegasys. **C.** Plasma from mice in **B.** was tested by p24 ELISA on the indicated days after infection. Each symbol represents an individual mice. *n* = 5 mice/group. For statistical analysis, non-parametric Kruskal Wallis test followed by Dunn's multiple comparison analysis was done. *p < 0.05; **p < 0.01.

To test the effect of pegasys treatment, we treated Hu-PBL mice with varying doses of pegasys and infected the mice 2 days later with HIV-1_BaL_ (20 ng p24). We repeated pegasys treatment on days 8 and 18 after infection and tested the mice for CD4 T cell depletion and plasma viremia. HIV infected (untreated) mice showed a profound depletion of CD4 T cells by day 10 after infection. This effect was prevented in a dose-dependent manner in the pegasys-treated group on day 10. However, even the highest dose treated mice started losing CD4 T cells by day 15, and by day 25, CD4 T cells were undetectable (Figure 2A, 2B). Plasma p24, which was reduced by day 10 in the pegasys-treated mice, also began to increase beyond day 15, and there was no difference between the control and treated group on day 25 (Figure 2C). Thus, pegasys treatment confers transient protection against HIV-1 replication in Hu-PBL mice.

### Hydrodynamic injection of plasmids encoding IFN-I leads to sustained IFN-I production and ISG upregulation

**Figure 3:**
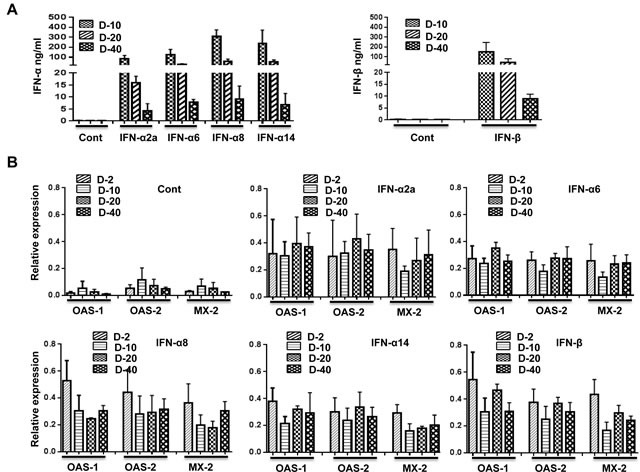

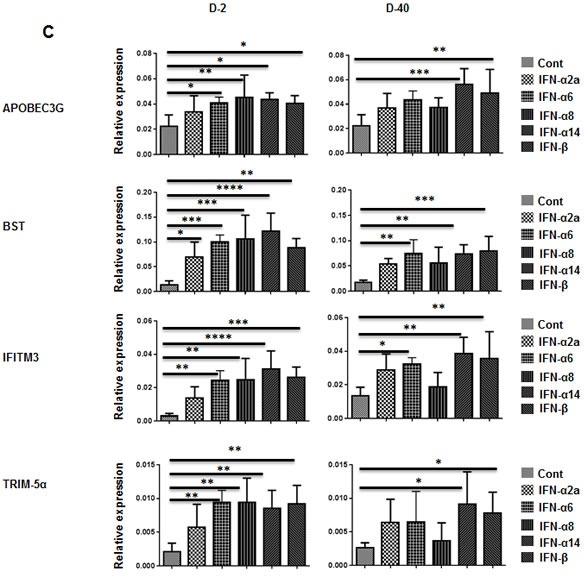
Injection of plasmids expressing subtypes of IFN-I results in prolonged upregulation of ISGs **A.** Hu-PBL mice were hydrodynamically injected with plasmids encoding IFN-α2, −α6, −α8, −α14 (left panel), or -IFN-beta (right panel) subtypes, and their sera were tested for IFN-I by ELISA at the indicated times after injection. *n* = 5 mice/group. **B.** PBMCs obtained on the indicated days after plasmid injection were tested for upregulation of the indicated ISGs over time by qRT-PCR. **C.** PBMCs obtained on days 2 and 40 after plasmid injection were tested for expression of indicated HIV-1 restriction factors by qPCR. Kruskal Wallis test followed by Dunn's multiple comparison analysis was used for statistical evaluation. *p < 0.05; **p < 0.01; ***p < 0.001; ****p < 0.0001.

Harper et al. recently reported an inverse relationship between IFN-α subtype expression and potency for reducing HIV-1 replication *in vitro*. IFN-α2 was highly expressed but was the least potent of the different subtypes in its anti-HIV effects. Therefore, we next wanted to test whether other subtypes enhance protection against HIV-1 infection *in vivo*. Human cytokines can be expressed in humanized mice by hydrodynamic injection of plasmids [29], which results in ready transfection of hepatocytes [30]. This method would also provide a possible means for gene therapy. Therefore, we first tested whether different IFN-I subtypes can be expressed in Hu-PBL mice. We rapidly injected (within 10 sec) 50 μg of plasmids encoding IFN-I subtypes in a 1- ml volume i.v. in groups of Hu-PBL mice and tested their sera for IFN-I at different time points by ELISA. All tested subtypes of human IFN-I were expressed in all mice at all time points tested (Figure 3A). The protein levels declined over time but were still detectable at substantial levels at the end point. We also tested ISG expression in PBMCs at different time points by qRT-PCR. Sustained upregulation of human ISGs (OAS1, OAS2 and MX-2) was seen for up to 40 days after injection (Figure 3B). Other HIV-1 restriction factors such as TRIM5α, TRIM22, SAMHD1, BST2 and APOBECC3G and IFITM3 have all been proposed to inhibit HIV-1, often with gene variant- or cellular context specificity[31]. Therefore, we also tested for induction of other HIV-1 restriction factors (APOBEC3G, BST, IFITM3 and TRIM-5α) on day 2 and 40 after plasmid injection. Although all these factors were upregulated in all IFN-I subtype expressing mice, in general, the levels were the lowest in IFN-α2 expressing mice and highest in IFN-α14 and IFN-β expressing mice (Figure 3C).

### Gene therapy with plasmids encoding IFN-β or IFN-α14 confers long-lasting protection against HIV-1 compared with other subtypes

**Figure 4:**
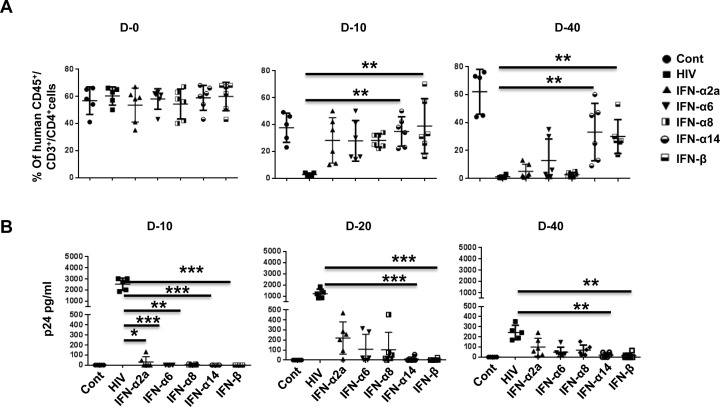
IFN-α14 and IFN-β confer prolonged protection against HIV-1 in Hu-PBL mice compared to IFN-α2 and IFN-α8 **A.** Hu-PBL mice were injected with plasmids encoding different subtypes of IFN-I and infected with HIV-1_BaL_ 2 days later. Blood collected on the indicated days after infection was tested for CD4 T cell depletion by flow cytometry. Data from 5 mice for CD4^+^ T cells as a percentage of CD3^+^ cells over time is shown. Each symbol represents an individual mouse. Cont, uninfected Hu-PBL mice; HIV, untreated HIV-1-infected mice; IFN, the indicated IFN-I plasmid expressing HIV-1-infected mice. **B.** Plasma from mice in (A) was tested by p24 ELISA on the indicated days after infection. Data from 6 individual mice are shown. For statistical analysis, non-parametric Kruskal Wallis test followed by Dunn's multiple comparison analysis was done. *p < 0.05; **p < 0.01; ***p < 0.001; ****p < 0.0001.

To test for an anti-viral effect, we transplanted human PBMCs into NSG mice, and after verifying human T cell expansion on day 10, we hydrodynamically injected the mice with plasmids encoding IFN-α2a, −α6, −α8, −α14, or −β. Two days later, the mice were infected with HIV_BaL_ (20 ng p24/animal) and tested for CD4 T cell depletion in PBMCs on days 10 and 40 and for plasma viremia on days 10, 20, and 40. Compared with uninfected mice, CD4 T cells were almost completely depleted on day 10 post infection in HIV-1-infected (empty vector injected) mice. By contrast, CD4 T cell depletion was prevented in all IFN-I subtype-expressing, HIV-1-infected mice by day 10 to a varying extent, most prominently in IFN-β- and IFN-α14-expressing mice. By day 40 after infection, complete depletion was seen in the control, IFN-α2a- and IFN-α8-expressing mice. By contrast, in the IFN-β- and IFN-α14-expressing mice, CD4 T cells were 50% preserved compared with control uninfected mice, and in IFN-α6-expressing mice, they were 25% preserved (Figure 4A).

Viral load was tested by p24 ELISA on days 10, 20, and 40. p24 was highest in control HIV-infected mice at all time points. A low level of p24 was detectable only in IFN-α2-expressing mice on day 10. On day 20, it was undetectable in IFN-β and IFN-α14-expressing mice but was detectable in the IFN-α2, IFN-α6, and IFN-α8expressing mice, although at a lower level than in infected control mice. On day 40, p24 was still barely detectable in IFN-β and IFN-α14-expressing mice but clearly detectable in the IFN-α2 and IFN-α8-expressing mice (30-40% of control mice, Figure 4B).

Taken together, our results suggest that, although all subtypes of IFN-I inhibit infection during early time points, certain subtypes, such as IFN-β and −α14 (which are not elicited during infection in humans [6]), have a robust and sustained antiviral effect. A recent study compared IFN-α2 with −α14 in BLT mice [32]. Similar to our results, they found that IFN-α14 effectively reduced HIV replication on day 11 post-infection. However, no effect was seen with IFN-α2, while we found that −α2 was also protective on day 10. This may be because in BLT mice IFN-α2 is induced endogenously due to infection, and further supplementation with exogenous IFN may not increase the protective effect, whereas in Hu-PBL mice, having no endogenous IFN-α2, the effect of IFN-α2 could be easily demonstrated. It would be interesting to test the combination of different subtypes for a potential additive/potentiating effect.

It should be noted that our study shows the effect of IFN-I on T cells. However, in the setting of a complete immune system, IFN-I subtypes have multiple and often complex effects on the innate and adaptive immune functions that could culminate in potentially beneficial or detrimental effects in chronic HIV-1 infection. In a recent study of SIV infection in rhesus macacques, blocking IFN-α signaling during only the first 4 weeks of infection resulted in accelerated disease progression and death from AIDS, suggesting a protective effect by IFN-α. However, treatment with pegasys (from 1-4 weeks post infection), although initially conferring resistance (in that it increased the number of intrarectal challenges needed for infection), also resulted in accelerated CD4 T cell depletion and increased viremia, probably due to desensitization induced by continued treatment [15]. By contrast, in the Hu-PBL mouse model, we found a sustained upregulation of ISGs for up to 30 days after plasmid injection, suggesting that IFN-α desensitization is not an issue in this model. Although the reason for this is not clear, it is possible that species-specific differences between monkey and human cells could account for this lack of desensitization. IFN-α has also been associated with HIV-induced immune activation in humans. However, we could not assess this effect in Hu-PBL mice because of the shorter duration of infection and the xenogenic activation of T cells in this model.

Overall, our results suggest that IFN-I has an important antiviral role during the acute stage of infection, but the effect of subtypes that are predominantly elicited by infection, such as IFN-α2, wanes over time. However, some subtypes that are not elicited during HIV infection, such as IFN-β and −α14, appear to have a robust and sustained antiviral effect, offering a potentially new therapeutic agent. Moreover, since prolonged expression of IFNs can be achieved *via* DNA injection, this could also provide a vector for gene therapy in treating HIV-1 infection.

## MATERIALS AND METHODS

### Animals

NSG were obtained from the Jackson Laboratory (Bar Harbor, ME) and maintained and bred in specific pathogen-free conditions at the TTUHSC animal facility, Paul L. Foster School of Medicine. All the experiments were performed with 6-8-week-old mice using study protocols approved by the TTUHSC IACUC committee.

### Hydrodynamic injection of plasmids expressing human IFN-α and IFN-β subtypes

Human IFNA-α2-expressing plasmid was obtained from Invivogen (San Diego, CA, USA). The human IFN-α6 (NM_021002.2), IFN-α8 (NM_002170.3), IFN-α14 (NM_002172.2), and IFN-β (NM_002176.2) genes were synthesized by Integrated DNA Technologies (Coralville, Iowa) and cloned into the pUNO plasmid (Invivogen). Plasmids were purified using the EndoFree Plasmid Maxi kit from Qiagen (Valencia, CA, USA). For expressing human IFN-β and IFN-α subtypes *in vivo*, human PBMC-reconstituted NSG mice were injected with 50 μg of plasmid using a 27-gauge needle in a total volume of saline equivalent to 8% of the body mass of the mouse, which was delivered within 10 seconds [33, 34].

### Human PBMC reconstitution and HIV infection

Human PBMCs were obtained from Astarte Biologics (Bothell, WA), and these cells (1×10^7^) were injected through the tail vein into 6-8-week-old NSG mice. Human cell reconstitution was analyzed by retro-orbital bleeding 10 days post injection. Animals were either injected with pegasys (on day −2, 8, and 18) or with IFN plasmids (on day −2). Animals were infected with 20 ng of R5-tropic HIV_Bal_ by the i.p. route on day 0 and were bled periodically through retro-orbital bleeding. Human cell reconstitution was analyzed by flow cytometry, and the collected sera were used for ELISA.

### ELISA and flow cytometry

IFN-α and IFN-β levels in mouse sera were determined using commercial ELISA kits (PBL Assay Science, Piscataway, NJ USA) according to the manufacturer's instructions. HIV-1 replication was analyzed by p24 ELISA assay (Perkin Elmer) according to the manufacturer's instructions. Various fluorescence-conjugated antibodies to human CD3, CD4, CD8, and CD45 were obtained from Biolegend (San Diego, CA) or Tonbo Biosciences (San Diego, CA). Cells were surface-stained using appropriate combinations of mAbs, and the samples were fixed and all flow cytometry data were acquired on FACS Canto II instrument (BD Biosciences) and analyzed with FlowJo software.

### qRT-PCR analysis

For determining the expression of ISGs following pegasys treatment, animals were injected with 200 ng of pegasys, and human cells were isolated 5, 10, and 15 days post injection. For determining ISG expression following IFN-1 plasmid injection, Hu-PBL animals were bled at 2, 10, 20, and 40 days post plasmid injection, and the isolated cells were used for RNA isolation. Total RNA was purified from the cells using the RNeasy kit (Qiagen), and cDNA was prepared using SuperScript^®^ III First-Strand Synthesis SuperMix (Invitrogen). qRT-PCR was carried out with the following primers: OAS-1 FW: TAGCTCCTACCCTGTGTGTGTG, OAS-1 REV: TCCTGAAAAGTGGTGAGAGGAC, OAS-2 FW: CTAGAGCAGATTGACAGTGCTG, OAS-2 REV: TCTTCAGAGCTGTGCCTTTG, MX-2 FW: CCAAGGACTTCAACTTTCTCAC, MX2- REV: GAGTCGATGAGGTCAATGCAG; BST FW: GACTATTGCAGAGTGCCCATG, BST REV: TGACATTGCGACACTCCATCA; TRIM5 alpha FW: GACGGAGAACGTGACCTTGA, TRIM5alpha REV: CCACTGTCACATCAACCCAGT; IFITM3 FW: CCTTCTTCTCTCCTGTCAACAGT, IFITM3 REV: ATGTGGATCACGGTGGACG; APOBEC 3G FW: TCTGGCTGTGCTACGAAGTGA, APOBEC 3G REV: TCCTCCACTTGCTGAACCAG;

GAPDH-FW: ATGGGTGTGAACCATGAGAAG, GAPDH-REV: TGGCATGGACTGTGGTCATG.

### Statistical analysis

Data were analyzed using prism 5.0 (GraphPad). For comparison of data with over two variables (IFN-α subtypes and IFN-β), non-parametric Kruskal Wallis test followed by Dunn's multiple comparison analysis was used. For data with two variables, non-parametric Mann-Whitney test was used. For all statistical tests, *P* value < 0.05 were considered significant. *P < 0.05; **P < 0.01; ***P < 0.001; ****P < 0.0001
